# Open Latarjet Procedure Using Double Suture‐Button Fixation

**DOI:** 10.1002/atn2.70182

**Published:** 2026-07-10

**Authors:** Graham Tytherleigh‐Strong, Caroline Atherton, Thomas Melton, Matthew Donaldson

**Affiliations:** ^1^ Division of Orthopaedics Addenbrooke's Hospital Cambridge University Hospital Trust Cambridge United Kingdom

## Abstract

The Latarjet procedure is the most commonly used technique for recurrent anterior instability with significant bone loss. However, the screws used to fix the coracoid are the major risk for postoperative complications including screw penetration and glenoid articular cartilage damage, prominence and impingement of the screw head onto the humeral head, and metalwork breakage. The use of suture‐button and cerclage fixation can significantly decrease this risk but has been primarily designed for arthroscopic use. We describe an open Latarjet technique using a modification of suture‐button fixation that does not require any additional specialized instrumentation. This allows surgeons who currently undertake an open Latarjet procedure to easily transition to suture‐button fixation with a minimal change to their established technique.

## Graphical abstract

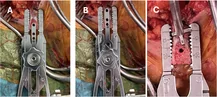

**VIDEO 1:** The video is of an open Latarjet procedure using double suture‐button fixation on a patient's right shoulder. This patient has had a previous failed Bankart procedure. The patient is in the beach‐chair position, and the coracoid process has been exposed through a standard deltopectoral approach. An osteotomy is performed using a curved osteotome, and the coracoid graft and conjoint tendon are mobilized. The undersurface of the coracoid is cur flat exposing cancellous bone, and it is loaded into the coracoid clamp. The drill guide is positioned over the center of the coracoid, and ×2 2.7 mm drill holes are made 10 mm apart. The distance from the drill holes to the edge of the coracoid is then measured at 7 mm. Having exposed the glenoid through a subscapularis split, a 7 mm offset tongues drill guide is positioned in the inferior quadrant of the glenoid. A 2.7 mm Beath pin is then drilled through the glenoid from anterior to posterior. As the Beath pin appears under the skin, posteriorly, a 2 cm longitudinal incision is made to allow the pin to exit posteriorly. A second Beath pin is then inserted in the same way using the tongued offset guide which has been positioned 10 mm above the first pin. A bullet dilating guide is then passed over each of the Beath pins posteriorly and pushed down onto the back of the glenoid creating a clear passage through the soft tissues. Suture buttons with their tapes are then loaded into the 2 drill holes in the coracoid. The sutures are then sequentially loaded into the eyelet of the corresponding Beath pin (inferior coracoid drill hole‐inferior Beath pin). The ends of the Beath pins are then pulled from where they exit posteriorly, shuttling the tapes through the glenoid posteriorly. Double eyelet buttons are then loaded onto the 2 suture loops for each suture button. A Nice knot is then thrown and tightened down onto the posterior glenoid for the inferior and then the superior suture button. At the same time, the position of the coracoid on the anterior glenoid is assessed and fine‐tuned. Once the correct position is obtained, the tails of the 2 sets of sutures are loaded into separate tensioners. The tensioners are then tightened to 100 nm. The inferior tensioner is then cycled 2 times to 100 nm, and the sutures are then tied securing the posterior button onto the glenoid. This process is then repeated for the superior tensioner. Video content can be viewed at https://doi.org/10.1002/atn2.70182.

The indications for using the Latarjet procedure have expanded over time to treat patients with significant bone loss and/or hyperlaxity, previous failed surgical stabilizations and as a primary procedure in high‐risk patients with subcritical bone loss. The standard technique uses 2 bicortical screws to fix the coracoid onto the neck of the scapular. However, although mechanically effective, screw fixation is the major source of complications (up to 30%) and the main reason for revision surgery and hardware removal (up to 10%).^1^

To minimize the effect of hardware issues and to adapt both the open Latarjet and Eden‐Hybinette procedures to an arthroscopic technique, a number of novel graft fixation methods have been developed. The most successful have been a suture‐button fixation technique (Smith & Nephew, Andover, MA, USA) and a suture‐tape cerclage fixation technique (Arthrex, Naples, FL, USA).^2^, ^3^ Both of these techniques use novel posterior drill guide systems and suture shuttling to insert the implants. Multiple studies have been undertaken to compare the biomechanical performance of these fixation methods and their clinical outcomes to screw fixation.^4^, ^5^, ^6^ In general, both the suture‐button and cerclage fixation techniques provided comparable fixation strength and clinical outcomes to screws, lessening the potential postoperative metalwork complications.^4^, ^7^

Despite this, in a recent study of 242 surgeons who routinely undertake Latarjet procedures, 98% used screw fixation and just 1% indicated cortical button use.^8^ However, 97% of the surgeons undertook an open procedure and only 3% of the surgeons used an arthroscopic technique. As the insertion technique for the suture‐button fixation system has been designed primarily for arthroscopic use, the probable reason that so many surgeons continue to use screw fixation is more to do with the technique than the performance of the suture buttons. Understandably, a surgeon who has developed a reliable open Latarjet technique, which is a challenging procedure, is unlikely to want to undergo the complex process of transitioning to an arthroscopic technique with its protracted learning curve.^9^, ^10^

We describe a simple modification to the current suture‐button system to undertake an open Latarjet drilling the glenoid from the front and not requiring a posterior drill guide or shuttling of the sutures.

## SURGICAL TECHNIQUE

The surgical technique is shown in Video 1.

### Patient Positioning

The patient is positioned in a three‐quarters beach‐chair position, with the back segment of the table removed, and prepared and draped exposing the posterior shoulder. This is to allow access to the back of the shoulder in order to load the posterior buttons and tie the sutures.  

**Step 1: Incision, Coracoid Osteotomy, and Coracoid Drilling**

An 8 cm longitudinal incision is made below the tip of the coracoid. The cephalic vein and the deltopectoral groove are identified. The vein is protected and retracted away from the groove, and the muscle interval is released and opened. The conjoint tendon is then released on both sides from the clavipectoral to aid mobilization. The coracoid is then exposed, and the coracoacromial ligament and the pectoralis minor tendon are completely released to skeletonize the coracoid.

We prefer to use a curved osteotome to perform an osteotomy of the coracoid at its base to obtain the maximum length. The coracoid is pulled forward carefully releasing the vascularized fatty tissue that is adherent to the undersurface of the coracoid and its junction with the conjoint tendon. The inferior aspect of the coracoid is decorticated to expose adequate cancellous bone.

**FIGURE 1:**
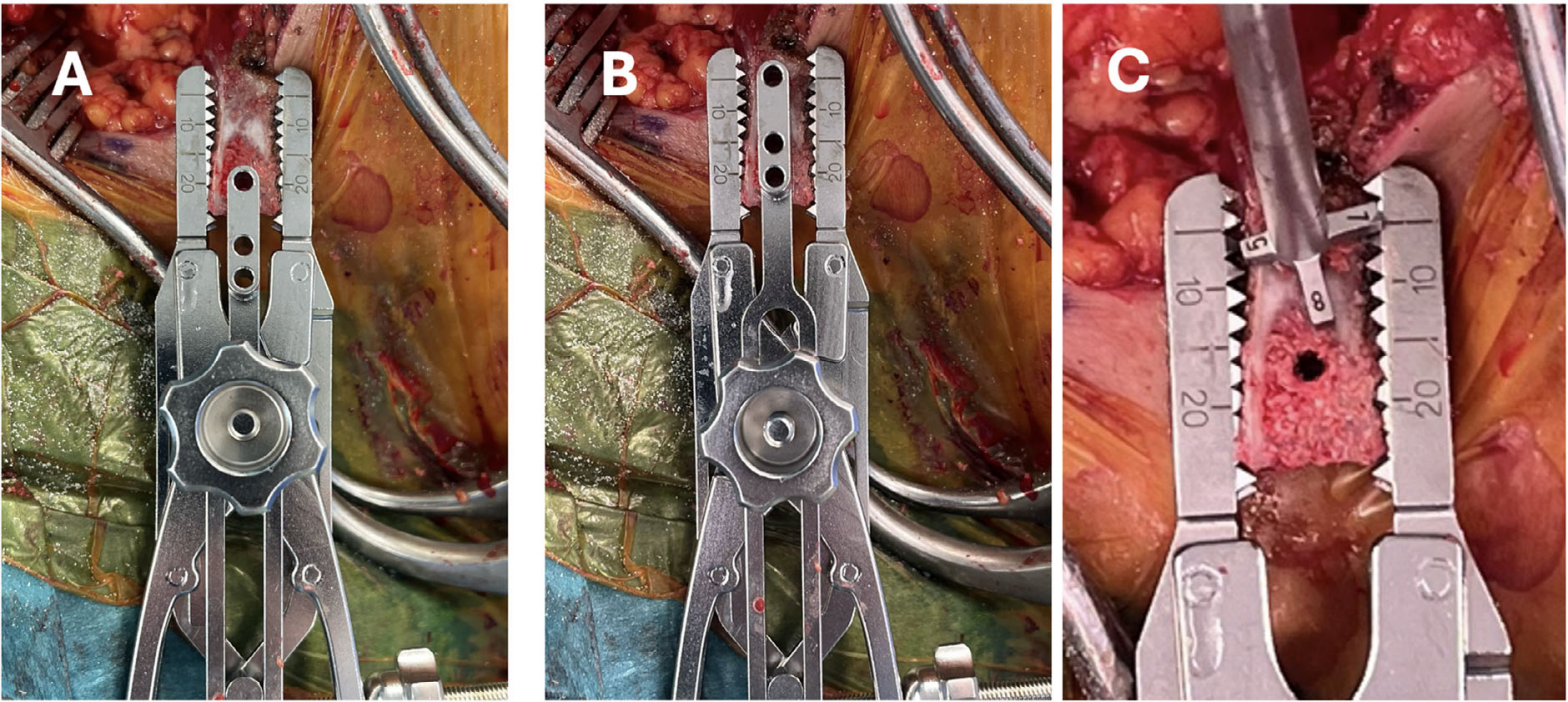
Coracoid clamp (Right Shoulder). (A) The inferior side of the coracoid has been prepared using an oscillating saw to remove the cortical bone, leaving a flat cancellous surface. The coracoid is held in the clamp with inferior surface upwards. (B) The central drill guide, with 2.7 mm holes that are 10 mm apart, has been moved and secured into the desired position for drilling the coracoid. (C) The coracoid measuring device has been placed in the inferior drill hole to measure the offset of the coracoid.

The coracoid is then loaded with the inferior cut surface upward into the custom bone block clamp (Smith & Nephew, Andover, MA, USA). This ensures that the drill holes will be perpendicular to the inferior cut surface. The drill guide arm is then rotated over the top of the bone block (Figure 1). The guide has two 2.7 mm drill holes 10 mm apart and can be moved up and down the length of the coracoid to find the optimal position for the drill holes. Once positioned, a 2.7 mm Beath pin is used to drill 2 holes through the bone block. The measuring guide is then used to size the width of the coracoid to calculate the offset for the glenoid drill holes.

**Step 2: Subscapularis Split, Glenoid Neck Exposure, and Preparation**

A retractor is used between the deltoid and pectoralis major muscle and the subscapularis muscle and tendon exposed. With the arm in adduction and external rotation, to apply tension, the muscle is split along its fibers just below the midlevel. The subscapularis muscle is then dissected off of the underlying capsule and a Gelpi retractor inserted. The capsule is then split from lateral to medial stopping at the edge of the glenoid. Sutures are then inserted into the inferior and superior leaves of the capsule to initially be used as retraction sutures and later as shuttle sutures to repair the capsule into a suture anchor at the end of the procedure. A vertical incision is made in the capsule at the joint line.

**FIGURE 2:**
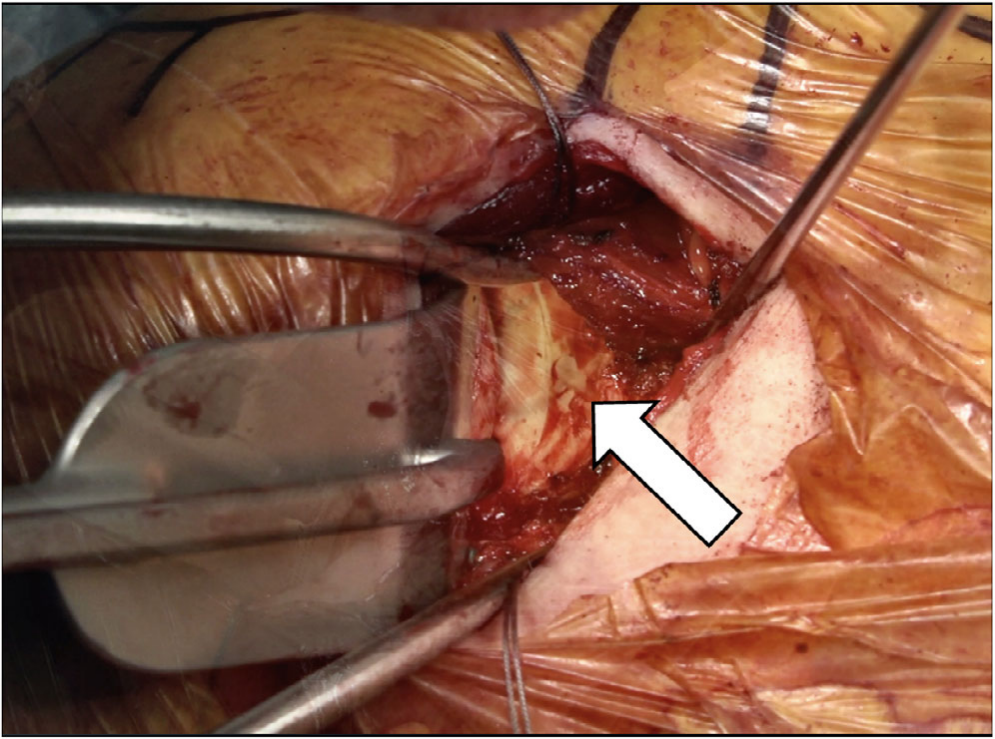
The glenoid has been exposed and the anterior surface has been lightly decorticated and flattened to provide a bleeding flat surface for the coracoid to be positioned and secured (white arrow) (Right Shoulder).

A Fukuda retractor with a light source (Smith & Nephew, Andover, MA, USA) is inserted into the glenohumeral joint to retract the humeral head laterally and the Gelpi retractor repositioned. A 3 mm Steinmann pin is positioned as medially and superiorly as possible underneath the superior split of subscapularis onto the scapular neck and drilled at an upward angle. This retracts the subscapularis superiorly. Having obtained adequate exposure, the capsuloligamentous tissue is dissected off of the anterior glenoid to expose the underlying bone. The bone is then lightly decorticated and a flattened surface to provide an optimal surface for bone contact and healing of the coracoid graft (Figure 2).

**Step 3: Glenoid Drilling, Loading of the Suture Buttons, and Passage of the Suture Buttons**

**FIGURE 3:**
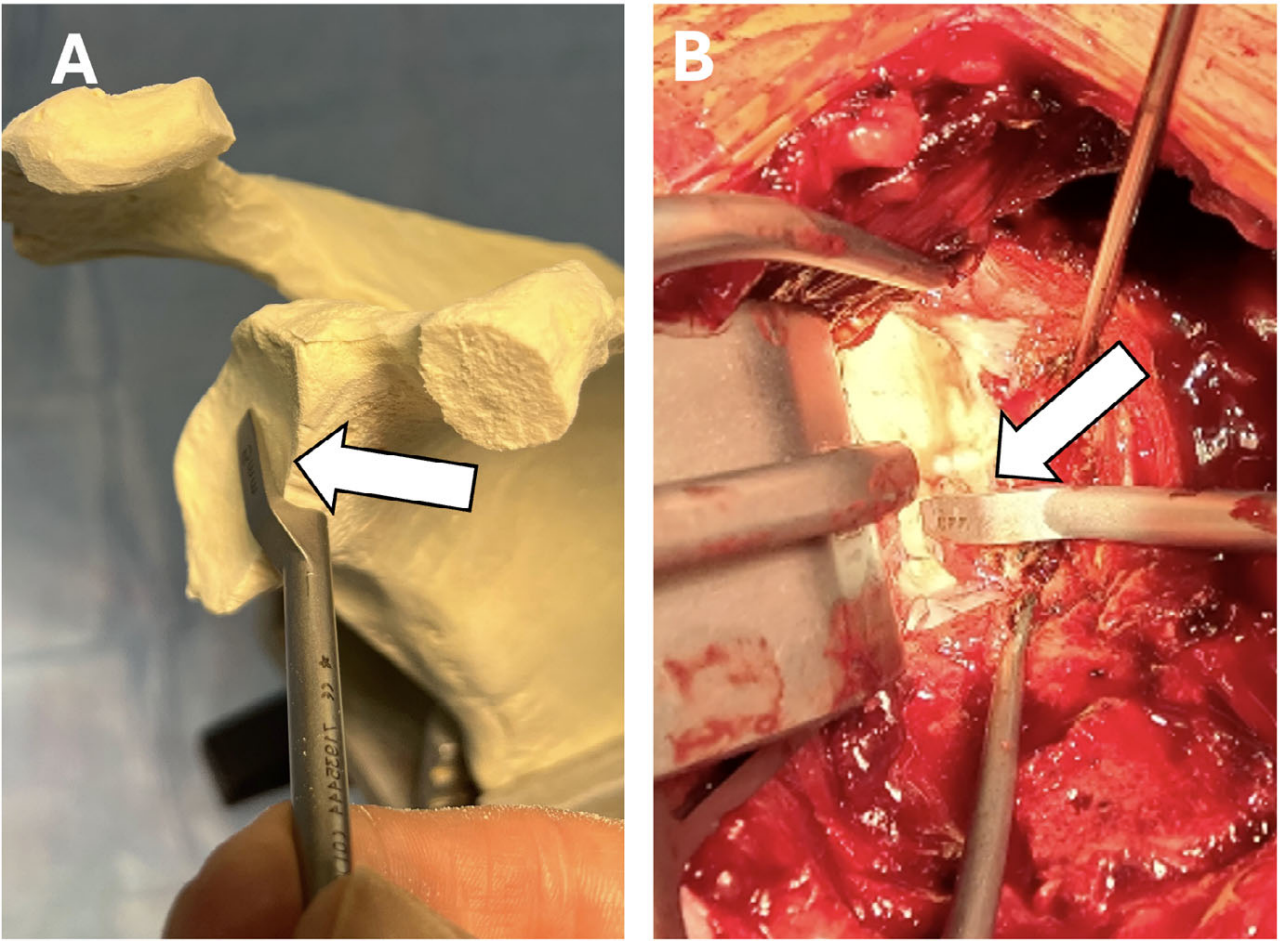
Tongued 10° 2.7 mm offset drill guide (Right Shoulder). (A) Saw‐bone right glenoid model showing a 7 mm offset guide positioned on the flattened anterior edge of the glenoid with the tongue flat on the glenoid articular surface (white arrow). (B) Intraoperative 7 mm offset guide positioned on the inferior anterior edge of the right glenoid (white arrow).

Having exposed and prepared the anterior inferior glenoid, a 10°, 2.7 mm, tongued drill guide (Smith & Nephew, Andover, MA, USA) at the previously measured coracoid offset is located at the position for the inferior glenoid drill hole (Figure 3). Confirming, with direct visualization, that the tongue of the guide is flat on the glenoid will ensure that the drill angle is within 10° of the axial plane of the glenoid avoiding damage to the inferior branches of the suprascapular nerve on the posterior surface.^11^ A 2.7 mm Beath pin is used to drill through the anterior and posterior cortices of the scapular neck and the posterior soft tissues. As the pin is about to penetrate the skin, a 2 cm longitudinal incision is made before it exits. The drill guide is then repositioned 10 mm above the inferior drill hole and a second Beath pin passed through the scapular neck and out through the skin posteriorly.

**FIGURE 4:**
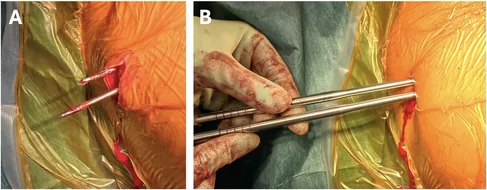
Posterior exit of Beath pins. (A) Skin incision over the posterior shoulder with the exiting 2 Beath pins 10 mm apart (Right Shoulder). (B) Bullet Dilating guides have been inserted over the Beath pins down to the posterior glenoid creating a clear passage for the posterior buttons to be inserted.

A Bullet Dilating guide (Smith & Nephew, Andover, MA, USA) is passed over both Beath pins and pushed through the posterior soft tissues down on to the posterior scapula (Figure 4). This creates a passage that will allow the posterior buttons to pass freely down to the posterior scapula, avoiding any tissue bridges when they are tied.

**FIGURE 5:**
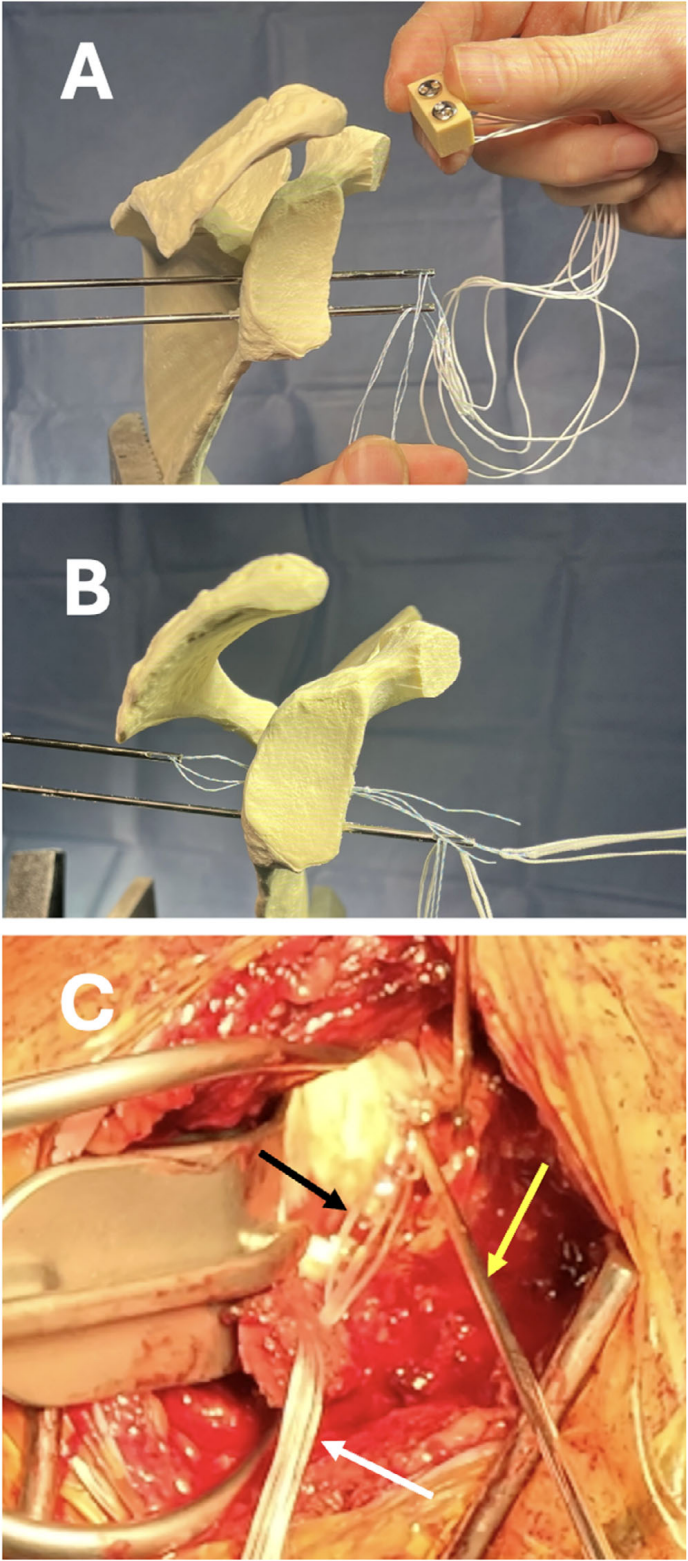
Shuttling the sutures. (A) Saw‐bone model of the right shoulder. The 2 Beath pins have been inserted from anterior to posterior through the inferior glenoid. The tails from the suture tapes have been loaded into the eyelets of the Beath pins. (B) The superior pin is being pulled posteriorly and has shuttled the tail from the sutures through the glenoid. (C) Intraoperative picture of the right anterior shoulder. The inferior Beath pin has been pulled out, and the sutures from the coracoid have been pulled through the glenoid (black arrow). The superior sutures can be seen coming out of the top of the glenoid (white arrow). They are about to be loaded into the eyelet of the superior Beath pin (yellow arrow).

Suture buttons are then loaded into the 2 drill holes that have been made in the coracoid from the superior to inferior surfaces. The ends of the sutures are then loaded into the corresponding eyelets of the inferior and superior Beath pins. Paying careful attention to suture management anteriorly, a pin puller is used to sequentially pull the Beath pins through the scapular neck and out of the skin posteriorly (Figure 5).

**Step 4: Loading of the Posterior Buttons, Graft Positioning, Tensioning and Tying of the Sutures, and Capsulolabral Repair**

Both sets of sutures from the inferior and superior buttons are then pulled tight, making sure that the sutures run freely. This will pull the coracoid onto the anterior glenoid.

**FIGURE 6:**
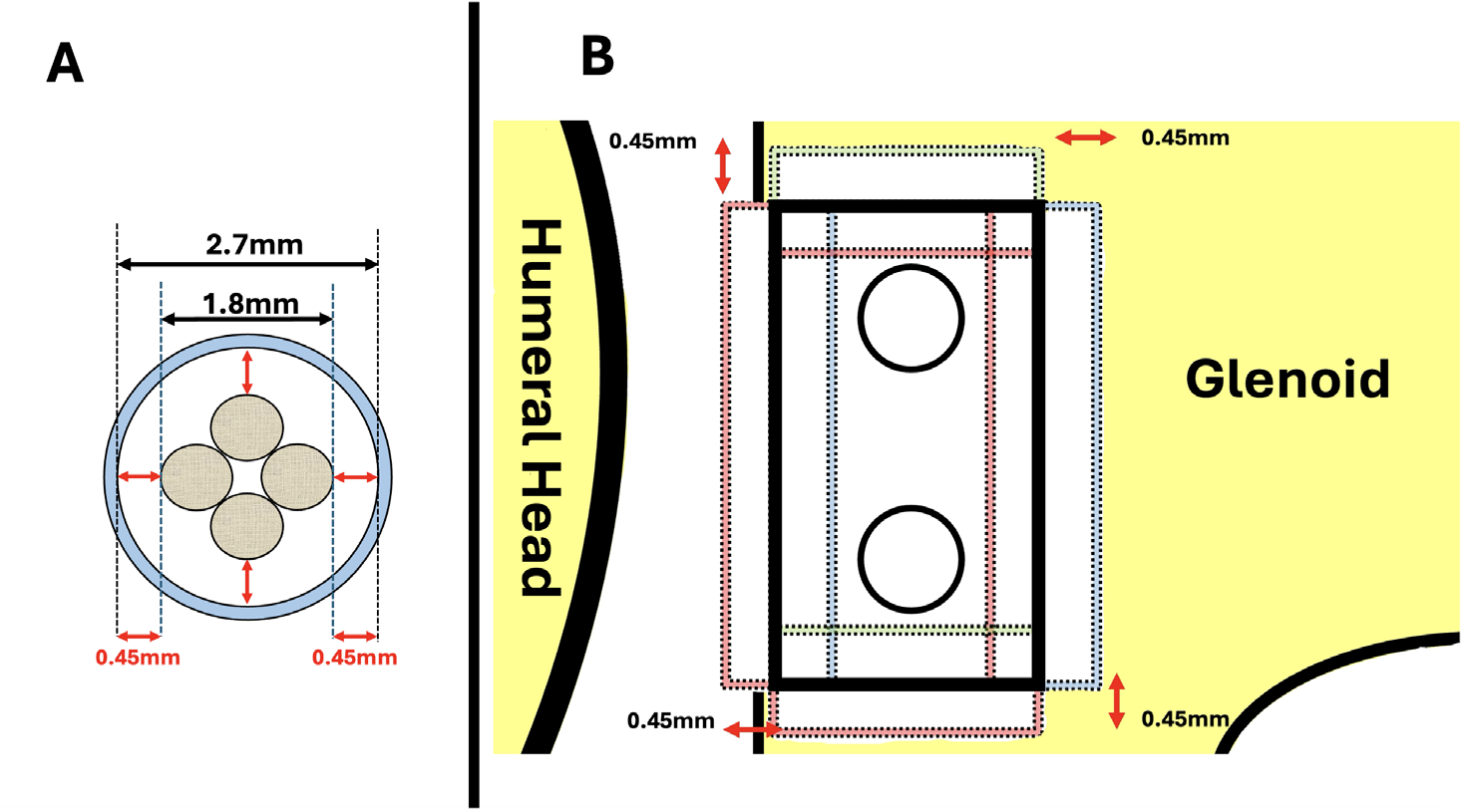
Flexibility of movement of the sutures within the glenoid drill holes (Right Shoulder). (A) Diagram of the relationship between the sutures within the glenoid drill. The diameter of the glenoid drill hole (blue circle) is 2.7 mm, and the effective diameter of the 4 sutures is 1.8 mm. This allows for a potential 0.45 mm displacement of the 4 sutures in the medial‐lateral and superior‐inferior directions (red arrows). (B) Diagram of the anterior glenoid and humeral head. The coracoid with its 2 drill holes (black rectangle) is in the centered position. Allowing for the potential movement of the sutures within the glenoid drill hole, the coracoid can be moved 0.45 mm medial (blue hash), lateral (red hash), superior (green hash), and Inferior (orange hash).

A double‐hole button is then loaded onto the 2 posterior suture loops of the inferior suture button. By pulling alternately on the suture loops, the sutures are cycled helping to break down any residual tissue bridges. A Nice knot is then formed with the 2 suture loops and pulled tight, pushing the posterior button down onto the posterior scapula. The remaining suture loop is then loaded into the tensioner. Before the tensioner is tightened, the position of the graft on the anterior glenoid is checked. As the drill holes are 2.7 mm in diameter and the effective diameter of the 4 tensioned sutures is 1.4 mm (No. 5 suture = 0.7 mm × 2) it is possible to adjust the position of the bone block both medial‐to‐lateral and superior‐to‐inferior by about 0.4 mm in each direction (Figure 6). Once the bone block is in a satisfactory position, the tensioner is tightened to 100 nm, to remove the creep (pretensioning) and left in position.

The above process is then repeated with a double‐hole button for the suture loops from the superior suture button. Once the tensioner for the superior suture button has been tightened to 100 nm, the tensioner for the inferior suture button is released, and 2 further cycles of 100 nm are then applied. To secure the knot (tensioning) and then to achieve bone‐block compression (overtensioning). This is repeated for the superior suture button. The stability of the bone block is confirmed, and 3 square knots are made to each suture to lock the construct.

The coracoid fixation is then inspected. If there is any lateral overhang of the graft over the joint, this is then carefully burred back.

**FIGURE 7:**
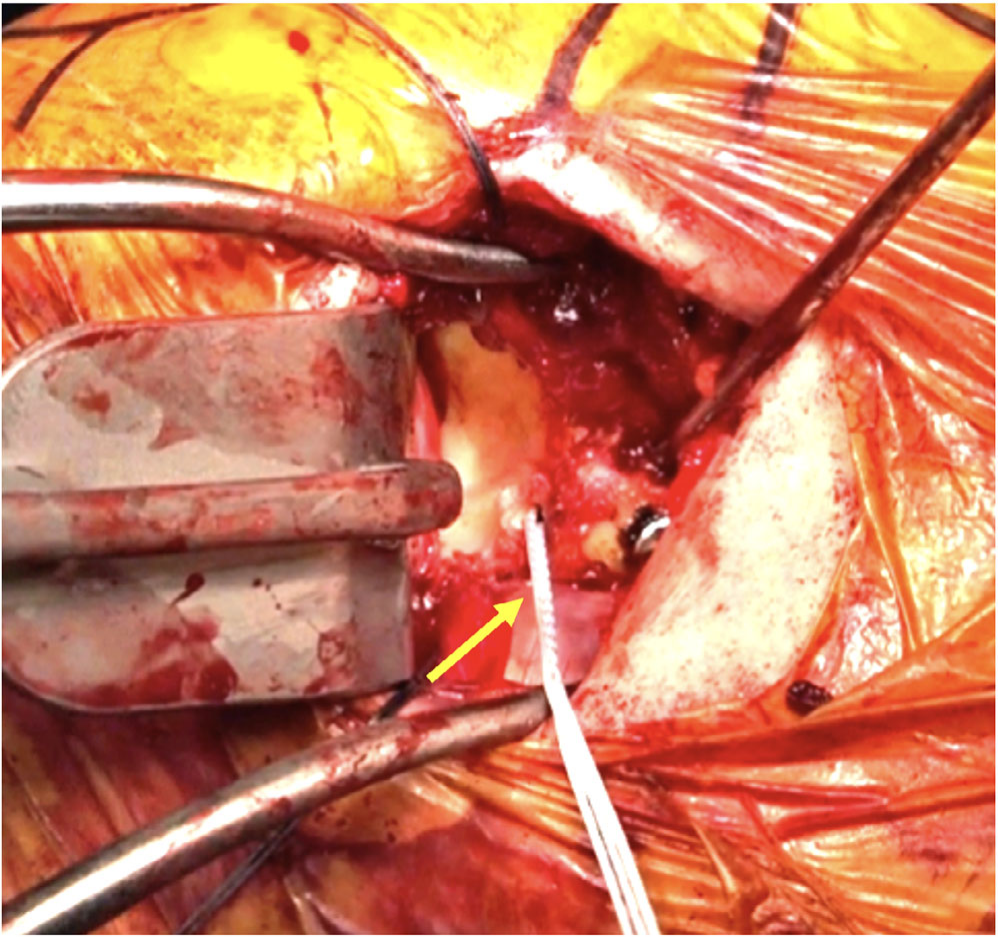
Completed procedure. The coracoid is securely fixed to the anterior inferior glenoid with no overhang, and a double‐loaded suture anchor has been inserted into the anterior glenoid between the inferior and superior buttons for the capsular repair (yellow arrow) (Right Shoulder).

For patients where there is still suitable capsular tissue present, a suture anchor is then inserted (2.9 mm Osteoraptor Suture Anchor, Smith & Nephew, Andover, MA, USA) into the anterior glenoid between the suture buttons behind the bone block (Figure 7). The sutures that had been inserted into the inferior and superior leaves of the capsule are used to shuttle the sutures from the anchor through the capsule. These sutures are then tied repairing the capsule, obtaining a superior shift and exteriorizing the bone block.

The tendinous lateral end of the subscapularis is then repaired to prevent the conjoint tendon from healing in the tendinous part of subscapularis which may inhibit postoperative external rotation. The wound is washed out and hemostasis achieved. The deltopectoral groove is approximated with 2 absorbable sutures and the wound closed in layers.

### Postoperative Rehabilitation

Postoperatively, the patient is kept in an immobilizer, limiting external rotation to 30°. At the 3‐week mark, they begin a standard Instability Rehabilitation program under the supervision of the physiotherapists.

At 6 to 8 weeks, they can use their shoulder without limitation and begin to start some closed‐chain strengthening exercises. From 12 weeks, they can return to a standard strengthening program and can commence noncontact activities. Between 4 and 6 months they should have regained a near full range of motion with 95% + return of strength. Over that period, they can begin a phased return for contact sports.

## DISCUSSION

Following the introduction of the Latarjet procedure in 1954, it has been considered the gold standard treatment for recurrent anterior shoulder instability with bipolar bone loss, with a reported recurrence rate as low as 1% to 3%.^12^ Its current indications are for the treatment of patients with significant bone loss and/or hyperlaxity, previous failed surgical stabilizations, and as a primary procedure in high‐risk patients with subcritical bone loss.

However, complications following surgery have been reported at a rate of 15% to 30%. These include nerve injury, graft mispositioning, osteolysis, nonunion, screw breakage, and prominence.^13^ Reoperation rates as high as 10% have also been reported. By far, the commonest reason for reoperation is related to screw malposition, screw prominence, and screw breakage.^1^

Patients with shoulder instability are at an increased risk of developing symptomatic osteoarthritis ultimately requiring a total shoulder arthroplasty.^14^, ^15^ In general, the relative number of patients with symptomatic anterior instability treated with a Latarjet procedure has increased.^16^ Although not considered a direct complication, the presence of 2 screws within the glenoid when undertaking a total shoulder arthroplasty will add to the complexity of the procedure.

In an attempt to decrease the complications and issues associated with screws, both in the short and long terms, and to improve graft fixation, multiple modifications to the instrumentation and implants have been described. Inserting the screws parallel to the glenoid articular surface minimizes any issues with screw head prominence, even in the presence of graft osteolysis. Various drill guides either placed directly onto the glenoid or through the coracoid have been designed. More recently, fixed‐angle hooked guides inserted into the back of the joint using a jig guide to drill from posterior to anterior have been introduced. These systems have allowed the use of either suture‐cerclage or suture‐button fixation. Bioabsorbable screws have shown an unacceptably high rate of graft osteolysis.^17^

Both the suture‐cerclage and the suture‐button fixation devices were developed for arthroscopic insertion using posterior guided drilling systems and suture‐shuttle techniques. The results for the suture‐button fixation used for an arthroscopic Latarjet have shown similar healing rates, time to return to sports, and functional results when compared with open screw fixation techniques.^3^, ^7^, ^9^, ^18^ A nonunion rate using suture buttons of 5% at 1 year post‐op has also been reported, which is better than the rates for screw fixation.^7^, ^19^

In practice, the arthroscopic Latarjet technique has never gained any real traction.^9^, ^20^, ^21^, ^22^ Currently, the vast majority of established orthopaedic surgeons who undertake Latarjet procedures use an open technique.^8^ However, while the potential benefits of a suture‐button or cerclage fixation over a screw fixation are known, their use has only been associated with an arthroscopic technique. As a result, most open Latarjet procedures still use screw fixation.

An open Latarjet technique has recently been described using suture‐cerclage fixation.^23^ This involves using the standard arthroscopic posterior drill guide introduced from the back of the shoulder, drilling the glenoid tunnels from posterior to anterior and shuttling the cerclage tape through the drill holes and glenoid tunnels. Essentially, this technique uses the same arthroscopic steps but as an open procedure.

The advantage of the open suture‐button technique that we have described is that it does not require any of the arthroscopic instruments that are used for the arthroscopic technique (Table 1). Its only real variation from a standard open Latarjet procedure is the use of suture buttons themselves and a Beath pin for the passage of the sutures. For surgeons who are considering converting from an open technique, there would be a minimal learning curve. The added advantage is that, because the drill holes in the coracoid are only 2.7 mm, at any point in the procedure, a surgeon could convert back to their standard screw fixation.

**TABLE 1:** Advantages and Disadvantages of the Technique

Advantages	Disadvantages
Safe and direct access for the coracoid osteotomy	Difficulty in performing the procedure in the lateral decubitus position
Comprehensive clamp and guide for drilling and preparing the coracoid	Open surgery is more invasive than arthroscopic surgery
2.7 mm coracoid drill holes allowing double fixation in smaller‐sized coracoids	Knot tying skills required
5‐8 mm offset guides to allow precise drilling of the glenoid tunnels	Need for small posterior incision
Ability to move the graft 0.45 mm superior‐inferior and medial‐lateral to “fine‐tune” the graft position prior to definitive fixation	
Tensioning devices to consistently obtain maximal graft fixation	
Easy transition from screw fixation to suture‐button fixation	
Option to overdrill coracoid and glenoid drill holes and convert to screw fixation	

There are also some additional benefits to this technique over the standard screw fixation: (1) The coracoid drill holes are only 2.7 mm in diameter, in comparison to 4 to 4.5 mm required for screws, which can decrease the issues of screw breakout that can occur with smaller coracoids. (2) The glenoid drill guide is placed directly onto the front of the glenoid which gives much better visualization and accuracy than using a guide inserted into the coracoid, which is then positioned onto the anterior glenoid. (3) The difference in the effective diameter between the 4 sutures and the glenoid drill holes allows for about 0.45 mm of movement of the coracoid both superior‐inferior and medial‐lateral to “fine‐tune” its position before tensioning and tying the sutures (Table 2).

**TABLE 2:** Tips, Pearls, and Pitfalls

Tips and Pearls	Pitfalls
Align the inferior surface of the coracoid flush with the drill guide to ensure that the coracoid drill holes are perpendicular to the decorticated surface	Make sure that the medial and lateral sides of the conjoint tendon have been adequately mobilized prior to undertaking the coracoid osteotomy
Insert a 3 mm Steinmann pin aimed toward the base of the coracoid and angled upward to act as a retractor for the superior part of the subscapularis split	Avoid excessive traction on the conjoint tendon to prevent a musculocutaneous traction injury
Obtain good visualization of the glenoid articular surface to ensure that the tongue of the drill guide is parallel before drilling the Beath pins	Remember to load the posterior button on to the sutures prior to tying the sutures and fixing the graft
Having loaded the suture tails into the Beath pins, pay attention to “suture management” to avoid any snaring or crossing of the sutures as they are pulled through the glenoid posteriorly	
Having passed the Nice knot and before applying the tensioner, make sure that the position of the coracoid graft on the anterior glenoid is optimal	

In summary, this modification of an open Latarjet technique using suture‐button fixation is a safe and reproducible procedure. The technique is a variation on a standard open Latarjet procedure and does not involve any complex new steps. As a result, it will allow surgeons who undertake an open Latarjet procedure to offer their patients the benefits associated with a suture‐button fixation.

## DISCLOSURES

The authors (G.T‐S., C.A., T.M., M.D.) declare that they have no known competing financial interests or personal relationships that could have appeared to influence the work reported in this article.
